# Does vegetation accelerate coastal dune erosion during extreme events?

**DOI:** 10.1126/sciadv.adg7135

**Published:** 2023-06-14

**Authors:** Rusty A. Feagin, Rachel A. Innocenti, Hailey Bond, Meagan Wengrove, Thomas P. Huff, Pedro Lomonaco, Benjamin Tsai, Jack Puleo, Maria Pontiki, Jens Figlus, Valeria Chavez, Rodolfo Silva

**Affiliations:** ^1^ School of Geography and the Environment, University of Oxford, Oxford, UK.; ^2^ Department of Ecology and Conservation Biology, Texas A&M University, College Station, TX, USA.; ^3^ Department of Ocean Engineering, Texas A&M University, College Station, TX, USA.; ^4^ Department of Civil and Construction Engineering, Oregon State University, Corvallis, OR, USA.; ^5^ Department of Civil and Environmental Engineering, University of Delaware, Newark, DE, USA.; ^6^ Instituto de Ingenieria, Universidad Nacional Autonoma de Mexico, Mexico City, Mexico.

## Abstract

A broadly accepted paradigm is that vegetation reduces coastal dune erosion. However, we show that during an extreme storm event, vegetation surprisingly accelerates erosion. In 104-m-long beach-dune profile experiments conducted within a flume, we discovered that while vegetation initially creates a physical barrier to wave energy, it also (i) decreases wave run-up, which creates discontinuities in erosion and accretion patterns across the dune slope, (ii) increases water penetration into the sediment bed, which induces its fluidization and destabilization, and (iii) reflects wave energy, accelerating scarp formation. Once a discontinuous scarp forms, the erosion accelerates further. These findings fundamentally alter the current understanding of how natural and vegetated features may provide protection during extreme events.

## Teaser

During storms and extreme events, vegetation accelerates coastal dune erosion.

## INTRODUCTION

Coastal sand dunes provide the first line of defense from storms for some of the most economically valuable and ecologically important landscapes in the world (*1*–*3*). A current and broadly accepted paradigm is that vegetation reduces the erosion of these dunes during wave attack (*4*–*7*). The existing body of dune research shows that a greater size (*8*), density (*9*–*11*), and diversity (*12*) of plants are associated with less erosion, although these studies have been limited to the investigation of relatively small wave events over short time scales (over minutes). Here, we show that during an extreme storm event, vegetation surprisingly accelerates erosion. While vegetation initially creates a physical barrier to wave energy, it also (i) decreases wave run-up, which creates discontinuities in erosion and accretion patterns across the dune slope, (ii) increases water penetration into the sediment bed, which induces destabilization, and (iii) reflects wave energy, which accelerates scarp formation. These findings fundamentally alter our current understanding of how natural features may provide protection during extreme events.

**Fig. 1.**
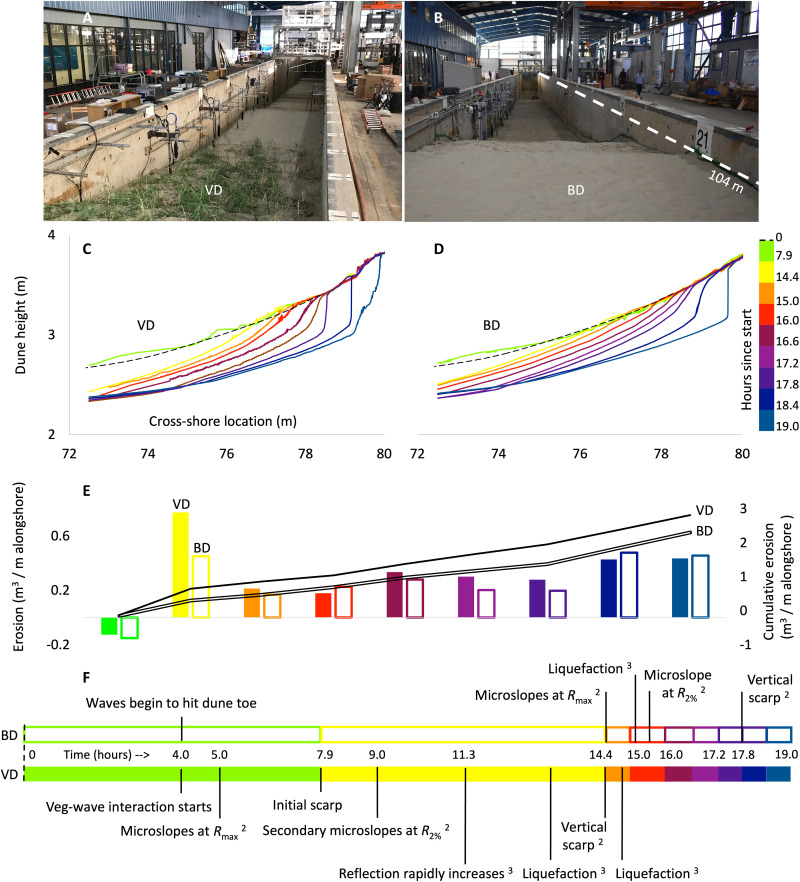
The dunes and their erosion during the extreme storm event. (**A**) Vegetated dune (VD). (**B**) Bare dune (BD). The different camera angles reveal beach berm and dune apex features, but the initial profiles for VD and BD were equivalent throughout the 104-m length of the flume. (**C**) VD profile evolution. (**D**) BD profile evolution. Initial profiles were equivalent at time zero as denoted by dotted lines (shown here only within the vegetated region from 72.5 to 80 m in cross-shore position). Colored lines depict the profiles at the end of the numbered time intervals. (**E**) Erosion during specified time intervals (bars) and cumulative erosion (lines). (**F**) Timeline (continuous) of major events in BD versus VD profile evolution. ^2,3^Refer to Figs. 2 and 3 for details.

To test the erosion of vegetated dunes (VDs) against bare dunes (BDs) in a controlled laboratory setting, where all other confounding variables could be removed, we constructed 70-m-long × 4.5-m-high beach-dune profiles and subjected them to waves in a 104-m-long flume. The profile geometry, sedimentary characteristics, and hydrodynamics were carefully constructed and monitored to mimic the progression of real storm events over time (Fig. 1, A and B; see Materials and Methods, figs. S1 to S7, and table S1). *Panicum amarum*, a typical dune plant common to North America, was grown in situ within the flume for 6 months before the experiment. The individual plant characteristics matched those encountered in the field, and the overall plant density and biomass were similar to those found on restored dunes or the lower portions of natural dunes that encountered frequent wave attack (figs. S8 to S10). Once the experiment began, we recorded the real-time evolution of the dune profiles (figs. S4 and S11 to S13) with an array of sensors and at 0.01-m spatial resolution with terrestrial laser scanners (TLSs).

## RESULTS

We found that both the VD and bare BD profiles evolved similarly during the early stages of a storm, until the waves first encountered vegetation, 4.0 hours into the event (Fig. 1, C and D). Shortly thereafter, VD formed an initial erosional scarp at 7.9 hours and had faster profile evolution as compared to BD. The vertical scarp was fully formed by 14.4 hours on VD, several hours earlier than on BD at 17.8 hours. By the peak of storm conditions, at 18.4 hours, the VD scarp was farther landward and nearly twice as high as that of BD (0.79 m versus 0.46 m in scarp height).

The final erosional volume, summed up until the peak conditions occurred, was 22% greater for VD compared with BD (Fig. 1, E and F). The cumulative erosion rates of the dunes diverged as the scarp began to form on VD at 7.9 to 14.4 hours, and there was over twice as much erosion on VD as BD by the end of this period (0.65 versus 0.30 m^3^ per meter alongshore). This difference was briefly narrowed just before 16.0 hours. Then, a second divergence in cumulative erosion rates occurred between 16.0 and 17.8 hours, after a fully vertical scarp was formed in VD and before any scarp formation in BD. However, once the BD scarp formation was underway, at 17.8 hours, the incremental erosional volume and cumulative erosion rates became more similar between VD and BD.

We identified three hydrodynamic mechanisms that led to the erosional differences between VD and BD. Each mechanism was altered by the presence of vegetation on VD and affected the other two through positive morphodynamic feedback.

**Fig. 2.**
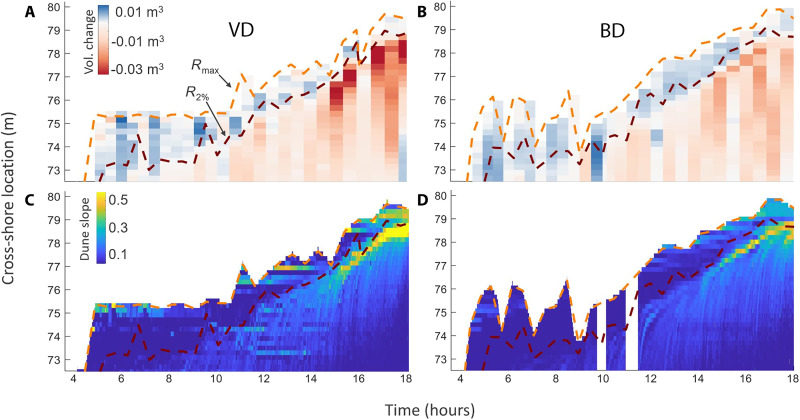
Wave run-up extent, accretion and erosion patterns, and micro-slope formation on the dunes over time. (**A**) VD maximum run-up distance (*R*_max_), *R*_2%_ run-up distance, and accretion-erosion patterns over the course of the storm. (**B**) BD *R*_max_ distance, *R*_2%_ distance, and accretion-erosion patterns. (**C**) VD micro-slopes over the course of the storm. (**D**) BD micro-slopes over the course of the storm. In (D), the white space around 10 and 11.5 hours on the *y* axis indicates data gaps. Axes for all graphics: The *x* axis ranges from the time that waves first encountered the vegetation (at 4 hours) until the peak of the storm conditions. The *y* axis ranges from the cross-shore location where the vegetation began at the dune toe (72.5 m) and extended landward to the dune peak (80 m).

First, we observed that the cross-shore excursion distance of the maximum wave run-up (*R*_max_) was over 10 times less variable for VD as compared to BD, during the first 11.3 hours (Fig. 2, A and B). For VD, the *R*_max_ distance was nearly constant during this time, intercepting the dune at a cross-shore distance of 75 m (SD = 0.08 m). For BD, the *R*_max_ distance ranged from 74 to 76 m (SD = 0.89 m). We also observed that the cross-shore excursion distance of the *R*_2%_ wave run-up (*R*_2%_ is the elevation that the largest 2% of all waves exceeded) was shorter for VD during this time, on average.

There was also more fine-scale variation in the VD slope at, and immediately offshore of, the *R*_max_ distance relative to BD (Fig. 2, C and D). Heterogeneous and localized slope discontinuities on the dune surface with a grade greater than 30%, or “micro-slope” features, first appeared at the *R*_max_ distance at 5.0 hours for VD. These first micro-slopes were colocated with mixed accretional-erosional hotspots, where individual run-up waves had removed sand from below *R*_2%_ and then deposited it before reaching *R*_max_ as they lost momentum and reached their cross-shore limit. By 11.3 hours, erosion seaward of both the *R*_max_ and *R*_2%_ lines began migrating shoreward and upslope as the storm progressed and the mean water level rose (as depicted by the lightening from dark blue to lighter blue colors, and eventually into micro-slopes, as depicted by the yellow colors). Micro-slopes were much less pronounced on BD, as the run-up and swash redistributed the sand over a broader range of the dune profile.

**Fig. 3.**
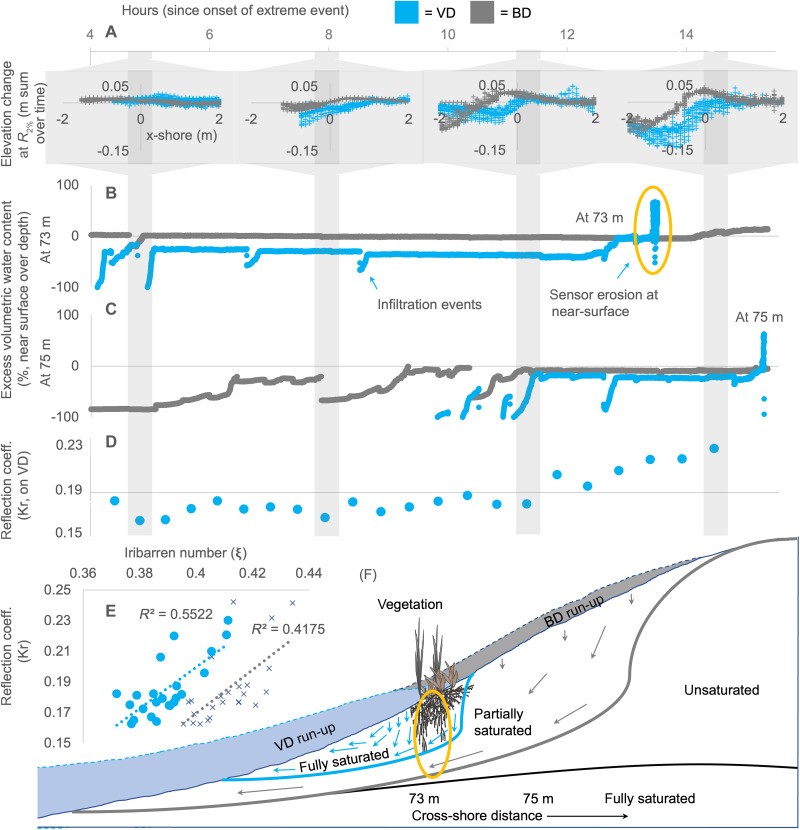
Dune erosion, water content and infiltration, and wave reflection over time. (**A**) Erosion and accretion at the *R*_2%_ line at 4.8, 7.9, 11.3, and 14.4 hours into the storm. The *x* axis for each subgraphic is the cross-shore distance from the *R*_2%_ line, and the *y* axis is the summed change per time period. (**B** and **C**) Excess volumetric water content across time, near surface at 73 m, and at 75 m in the cross-shore direction, respectively. The *x* axis is continuous time in hours. Positive values on the *y* axis refer to near-surface volumetric water content exceeding that at depth, negative values vice versa. (**D**) Wave reflection across time highlighting the apparent transition at 11.3 hours on VD. (**E**) Wave reflection for VD and BD as a function of the Iribarren number; VD = 1.4*x* − 0.4, *R*^2^ = 0.42, blue circles; BD = 1.3*x* − 0.3, *R*^2^ = 0.55, black crosses. (**F**) The vegetation retarded wave run-up and concentrated water volume, supersaturating the sand and promoting instability.

Second, the vegetation on VD (and its induced micro-slopes) concentrated the run-up water volume into a smaller volume of sand, which precipitated greater profile steepening and micro-slope instability (Fig. 3, A to C). At least initially, the consistently retarded run-up distances on VD kept the sand in the vegetated region of VD drier than that of BD, for the same cross-shore location. However, once the run-up passed this location and was retarded farther upslope, the water rapidly infiltrated and the volumetric water content increased on VD. In effect, the run-up water volume had been concentrated into a smaller volume of sand by the vegetation. Once the volumetric water content at the near surface began to exceed that at greater depths, the dune sands were increasingly poised for instability (see figs. S12 and S13).

Third, wave reflection was increased by the vegetation and its induced micro-slopes (Fig. 3, D to F). As soon as the waves intercepted the vegetated region of VD, at 4.0 hours, offshore wave reflection became greater on VD than on BD. Throughout the evolving storm conditions, the reflection was consistently 3% higher on VD (across the varying dune slope, wave height, and wave period combinations, as recorded by a range of Iribarren numbers, ξ = 0.37 to 0.44 among both VD and BD; linear regression fit for each as VD = 1.4*x* − 0.4, *R*^2^ = 0.42; BD = 1.3*x* − 0.3, *R*^2^ = 0.55).

Silva *et al.* (*9*) attributed a similar 4% increase in reflection to vegetation for ξ = 0.4, due to increased friction within the water column, but did not relate it to increased erosion. Reflection rapidly increased at 11.3 hours, as the micro-slopes accumulated and the scarp quickly steepened.

**Fig. 4.**
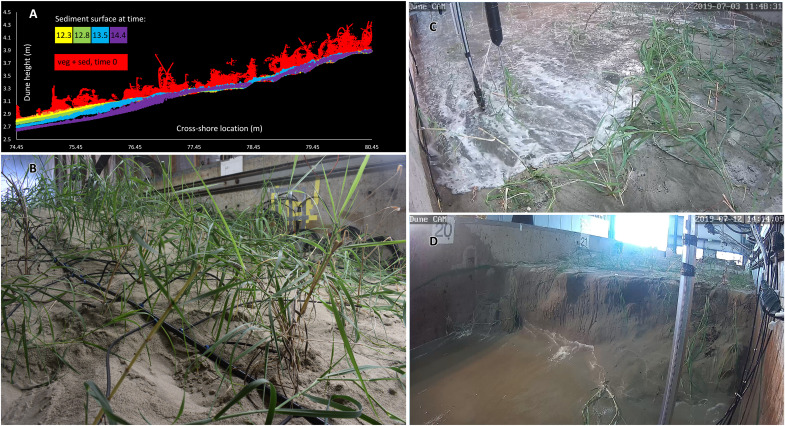
Morphologic changes on VD during the extreme storm event. (**A**) An example of Terrestrial Laser Scanning (TLS) data for the VD profile, viewed from the cross-shore dimension (i.e., the side), at 0, 12.3, 12.8, 13.5, and 14.4 hours into the storm conditions. The red points depict both the vegetation and the sediment surface before the experiment began at 0 hours. The other colors depict the sediment surface only, between 12.3 and 14.4 hours. By 14.4. hours, an initial 0.1-m tall scarp was apparent at ~76.5 m. (**B**) *P. amarum* plants on VD before the experiment began at 0 hours. Drip line (black hose) was removed during experimentation. (**C**) Run-up intercepting plants on VD at 11.3 hours. The depicted sensor array included an optical sediment gauge, sediment movement sensor, ultrasonic sensor, and acoustic velocimeter. (**D**) A 0.79-m tall scarp on VD at 18.4 hours.

By 14.4 hours into the storm, a small and initial scarp 0.1 m in height was formed on VD (purple line at 76.5 m in Fig. 4A, and as also shown as the transition from bright green to yellow colors at ~76.5 m in Fig. 2C), and erosion accelerated as the beach-dune profile transitioned from a swash regime to a wave collision regime (Fig. 4, B to D). The dune was then undercut laterally, and the gravity-driven processes of scarp slumping began (*13*, *14*), causing erosion in relatively large failure events. By this time frame, the eroded sediment had begun to accumulate into a ~6-m-wide underwater bar within the flume, with its peak ~28 m from the original dune toe. The waves were then more hindered by water depth limitations and impacted the profile with greater prior energy dissipation. This negative feedback loop of scarp slumping, nearshore bar growth, and decreased-intensity wave impact happened earlier on VD, potentially explaining how VD and BD became more similar toward the end of the experiment.

In our experiments, transition to the collision regime on VD began at 7.9 hours and there was a fully vertical scarp by 14.4 hours. In contrast, a wave swash regime existed on BD for much longer, with a transition to collision occurring at 15.5 hours and with a fully vertical scarp at 17.8 hours. In sum, the collision regime on VD persisted for a longer period of time than on BD (11.1 hours versus 3.5 hours, before the peak of the storm conditions), accounting for the greater cumulative erosion.

## DISCUSSION

On the basis of the principal finding of this study, coastal engineers and managers may need to reexamine the predominant paradigm that dune vegetation reduces erosion during extreme events. As previous literature (including our own work) suggests, vegetation initially reduces the erosion caused by small waves over short time scales (minutes). However, as we show here, on planted and restored dunes, vegetation actually accelerates erosion over the course of an entire storm by inducing an earlier transition to a collision regime. In essence, dune vegetation disrupts the morphodynamic balance, tipping it toward the creation of micro-slopes and vertical instability.

Accordingly, engineers may need to further explore the assumptions that support models such as XBEACH (*15*) and CSHORE (*16*), which are used to design shoreline protection projects around the world (*17*). In these models, the vegetative roughness-induced friction and drag at fine spatiotemporal scales (e.g., *18*–*20*) have been assumed to raise the water velocity threshold required for sediment mobilization to begin. While this effect reduces erosion to the aft of a plant, the broader concept is that vegetative structure can also induce scouring and supersaturation in front or to the side of the plants (*21*), creating spatially heterogeneous accretion and erosion patterns (*5*) and micro-slopes. To progress with these models, fine-scale physics and hydraulics must be better synchronized with the morphodynamic tipping points that delimit the erosional regimes of Sallenger (*13*)—without the assumption that vegetative structure always reduces erosion at coarser time scales.

Still, we contend that the value of planting vegetation for coastal protection can be gained in the years to decades before a single storm event—and this value is related to the ecological function of the plants rather than their biophysical structure alone (*22*). While sand can accumulate around any living or nonliving structure, native dune plants grow in size, replicate, and are stimulated by burial. Moreover, the linked ecogeomorphic process of succession acts at the landscape scale over longer time frames to control the shape of the dunes (*23*). The unique ecological function of living materials includes the ability to physiologically adapt to dynamic storm conditions, create spatially heterogeneous erosion and accretion patterns with a diverse array of plant species that fill several niches at the landscape scale, and continue to migrate landward and provide ecosystem services as the sea level rises globally—all of which help build the dunes. If vegetation is absent or unhealthy over the years before a storm, the dunes will be smaller when the storm hits and the damage to landward locations will be greater.

Historically, many coastal managers have assumed that the physiological aim of vegetation is to stabilize the sand during extreme events (*24*). Our results provide initial evidence that this assumption is incorrect, and we contend that the assumption has had negative consequences for coastal protection efforts. For example, after the 2004 Indian Ocean tsunami and the 2011 Tōhoku tsunami, nongovernmental organizations (NGOs) and government agencies planted nonnative *Casuarina equisetifolia* trees across large areas of the coast, believing that their structure will protect the coast during future extreme events. However, these policies ultimately increased erosion over longer time scales by replacing native plants, which led to less dune accretion and lower elevations (*24*). A similar story has played out with the use of various *Ammophila*, *Spartina*, *Oenothera*, and *Tamarix* species for stabilization projects around the world over the past century [e.g., (*25*)]. As shown here, an attempt to stabilize a dune based on a narrow view of vegetative structure will only accelerate its loss during an extreme storm event. Rather, coastal managers should select an appropriate species based on their ecological function to build the dunes in the years before the event occurs.

Although we tested the effect of only one species here, *P. amarum*, there is no reason to believe that the results would be qualitatively different given another species. Fundamentally, any living or nonliving structure that is placed onto the beach-dune profile will create a discontinuity in wave run-up and energy flow across the landscape. The same three mechanisms of enhanced erosion that we have discussed here will apply, and in fact are likely to be amplified in a case of an even greater plant coverage, biomass, or physical stature.

Moreover, as has been shown in the past by other dune studies, aboveground and belowground stems, stolons, and leaves function similarly under wave attack (*5*–*6*) and only fine roots hold onto the sediment in a unique manner (*7*). Thus, if anything, VD likely yielded an underestimate of the erosion caused by vegetation on the upper portions of real-world dunes covered by *P. amarum* (as VD was high in fine root biomass, yet low in other types of biomass, as compared to field conditions—see Materials and Methods). For real-world dunes, the key portion of the morphologic sequence that is most affected by vegetation is during the run-up regime and the initiation of scarp formation, which most typically occurs on the lower portions of the dunes where the vegetation densities are relatively lower. For these reasons, we expect that our findings are universalizable in a qualitative manner across all VD types, when they are subjected to extreme events over hours to days of time. We expect that only the absolute quantities of vegetation-induced erosion may differ as based on the specific species or planting densities.

Notably, our experiments did not continue beyond the peak of the simulated storm conditions. We can speculate, however, that the cumulative erosion differences should persist in time for real-world dunes, even after a storm, as the water level draws down and the interaction with vegetative structure ceases. We also note that the induced storm conditions were not designed to approximate a given spectrum of infragravity motion at dune toe, but rather to match the spectrum within the inner surf zone, based on a modeled dataset (figs. S1 and S2). However, wave transformation and reflection between these two positions in the bounded flume likely inserted somewhat similar motion, and the *R*_max_ and *R*_2%_ statistics appeared to be generally consistent with real-world datasets.

In summary, we found that the net effect of planted dune vegetation was to increase erosion during extreme events. Vegetation creates a physical barrier that shortens the length of maximum wave run-up, alters accretion and erosion patterns, and creates steep micro-slopes. Compared to bare dunes, vegetated dunes concentrate the same volume of water into a smaller volume of sand, which then predisposes the dune to fluidization and micro-slope formation, wave reflection, earlier scarp formation, and more rapid dune erosion over the course of a storm.

## MATERIALS AND METHODS

### Hydrodynamic conditions

We sought to investigate the hydrodynamic conditions experienced along a nearshore-beach-dune profile during an extreme storm event, as the water level rose, the storm-induced wave energy increased, and the dune began to erode. Thus, the hydrodynamic conditions were designed to match those generated by a typical tropical cyclone as it moved toward the coast, with peak conditions at 18.4 hours. We primarily based these conditions on measured and modeled waves during Hurricane Sandy in 2012 (fig. S1). We obtained the heights and periods 680 m offshore of Mantoloking, New Jersey using WAVEWATCH III (*26*) and the water levels 140 miles offshore using National Oceanic and Atmospheric Administration (NOAA) buoy #8534720 (*27*).

However, we also modified these conditions to be representative of a broader range of typical storms and rescaled them [e.g., (*28*–*30*)] to fit the inner surf zone (fig. S2 and table S1). The experiment was focused on correct scaling within this inner surf zone and landward. The scaling was also informed by past flume experiments for dune erosion within the same facility (*31*). The following field conditions were used for scaling analysis: bore height in the inner surf zone after offshore dissipation (*H* = 0.6 to 1.0 m); wave period (*T* = 4.5 to 7 s), inner surf zone depth (*h* = 2.1 to 2.8 m); median grain size (*d*_50_ = 0.21 mm); and an undistorted geometric scale from the berm landward of 1:1 (field:laboratory). These considerations indicated a Froude (time) scaling of 1 and a sediment grain size scaling of 1 based on the undistorted inner surf zone depth ratio [e.g., a flume grain size of 0.21 mm; (*28*)]. The Reynolds number, *Re,* was ~7 × 10^6^ under field conditions (*U* ~ 3 m/s; *h* near the dune ~ 0.25 m). *Re* decreased by 40% (*U* ~ 2 m/s) for the laboratory conditions but was still fully turbulent, a criterion suggested to be sufficient for laboratory simulations (*28*). The Shields number was used to determine transport mode and was ~2.25 times larger in the field (*30*), but both values exceeded the critical value (θ ~ 1) when sheet flow conditions were expected to occur (*29*). Live vegetation was used and roughly matched field conditions (see text below under the “Vegetated conditions” section) such that there was no vegetation drag scaling. In summary, the fundamental hydrodynamic forcing and processes of sediment and vegetation response were well represented.

We then created a Texel-Marsen-Arsloe (TMA) shallow water spectrum (*32*) in the 104-m-long O.H. Hinsdale Large Wave Flume (LWF) in Corvallis, Oregon, and slowly increased the intensity of the storm conditions over time to match these inner surf zone characteristics. This facility is further documented in (*33*). This flume is the largest in the United States and is a key facility as part of the U.S. National Science Foundation’s (NSF) Natural Hazards Engineering Research Infrastructure (NHERI) program.

For the experiment, the average significant wave heights ranged from ~0.6 to 1.0 m, periods from ~4.5 to 7.0 s, and water depths from ~2.1 to 2.8 m. Storm waves that arrive within 100 m of the subaerial beaches and dunes are typically within these height and length scales, due to depth-limited wave breaking and self-similarity of wave decay of larger offshore waves as they propagate through the nearshore (*34*). In other words, the waves that struck the dune profiles were of the same magnitude as those found in the field.

### Dune profile construction

As the hydrodynamic conditions progressed over the storm, we recorded topographic (subaerial) and bathymetric (subaqueous) changes along vegetated and bare nearshore-beach-dune profiles. These profiles were designed to match those of a typical beach and were roughly modeled on a profile measured in the field at Mantoloking, New Jersey (figs. S3 and S4). The sand (median diameter = 0.21 mm) was collected from South Beach, Newport, Oregon, and over 500 m^3^ (or about 100 standard dump truck loads) was placed into the LWF (figs. S5 and S6).

VD and BD were similar in profile geometry and compaction before encountering the storm conditions (fig. S7). We measured compaction at the surface and at depth at three locations on the dunes, using a dynamic cone penetrometer (K-100 DCP, Kessler Soils Engineering) before each experiment. We found similar sediment compaction values for both dunes (and they were both similar to values recorded at the field site sand source). The only notable trend was that the surface sediments were generally looser than those at depth, for both dune types.

We placed a large number of sensors on and around the dune profiles to record the conditions. The sensors included a large number of capacitance wave gauges, ultrasonic sensors, acoustic Doppler velocimeters, volumetric water content sensors, accelerometer sensors, pore pressure sensors, drag force sensors, turbidity sensors, sediment movement sensors, optical sensors, and video cameras among others (only sensors relevant to this article are shown in fig. S3).

The sensor locations were surveyed using Total Station, and all recorded data were placed into a common coordinate system. The neutral position of the flume wave maker was referenced as 0 m in the cross-shore direction, and the bottom position of the wave flume was 0 m in elevation.

### Vegetated conditions

Our goal was to best match the typical vegetative conditions that can be found on real-world dunes, although we acknowledge that such dunes are complex structures that can take years to decades of time for ecogeomorphic processes to appropriately build.

The plants (*P. amarum*) for the VD profile were collected from Galveston Island State Park, Galveston, Texas, and were grown in situ at the LWF for 6 months, across an area of 43.9 m^2^, at a 0.5-m spacing (cross-shore locations, 72.5 to 84.5 m). Initially 1000 nodal cuttings were first propagated in a greenhouse in Corvallis, Oregon for 1 month in January 2019. Of these, 168 plants, healthy and of a similar size, were then planted directly into the LWF profile and grown for another 5 months, with a staggered spacing of 0.5, such that every-other row of vegetation was aligned in the cross-shore direction (figs. S8 to S10). Plants in the flume were grown using an automated drip irrigation and lighting system, and were monitored for temperature, humidity, and soil temperature. They were fertilized in February and April at 90 kg/ha using 12-4-8 standard liquid fertilizer.

After 6 months of growth, there were no distinct trends in the aboveground biomass in the cross-shore or along-shore direction. Similarly, there were no trends in the wet belowground biomass across the dune, with depth, nor immediately below a plant versus around the plant spacing gaps. The vegetation density was 3.83 plants/m^2^ for the experiment (*n* = 168 plants divided by area they covered 43.98 m^2^).

At the start of the experiment in June, the individual aboveground characteristics of the plants in the flume matched those from previous field work (*5*) in terms of mean plant height, stem diameter, and elasticity. In terms of aboveground plant material, the mean stem diameter of the plants in the experiment was 0.55 cm (*n* = 15 samples) versus 0.6 cm in Texas plants and 0.5 cm in North Carolina plants from the field. The mean stem height was 30.67 cm (*n* = 15) versus 43.2 cm in Texas plants and 31.7 cm in North Carolina plants from the field (*5*). The mean Young’s modulus of elasticity (*E*) for the plant stems was similar to that found in (*20*): 0.043 ± 0.036.

For belowground plant material, we measured an average of 861.1 ± 741.4 g/m^3^ in wet belowground biomass using 28 soil cores that were 10.5 cm diameter by 60 cm depth that were extracted from the peak of VD (collected before the experiment, never altered by the hydrodynamics). This material was nearly all fibrous roots, and this value was greater than that found for the fibrous roots only in the field in North Carolina at 320 g/m^3^. We did not have a field value for Texas plants. However, the total belowground biomass, to include buried stems and decomposing leaves, was lower than the 1955.7 g/m^3^ in North Carolina plants and 2704.0 g/m^3^ in Texas plants in the field (*5*). In the case of the flume-based VD, there were no buried stems and decomposing leaves, rather only fibrous roots.

Visually, the overall plant density and aboveground biomass appeared lower than those of a natural dune, but similar to those of a restored dune or the lower embryonic portions of a natural dune where wave attack is most frequent (Fig. 4 and figs. S8 to S10).

### Profile morphology

To record the morphologic evolution of the profiles, we scanned their subaerial portions immediately after intervals of wave attack at a spatial resolution of 0.01 m (Fig. 4A and fig. S11). We scanned from a fixed and constant point with a Leica ScanStation2 Terrestrial Laser Scanning (TLS) system (Leica Geosystems Inc.), used targets to co-register the scans, and extracted the datasets using Cyclone software (Leica Geosystems Inc., v.6.0.3). For VD, we used the segment tool in CloudCompare (GitHub open source, v2.11) to manually remove the vegetation and identify the underlying sand topography. Additionally, we scanned the subaqueous portion of the profile with a sonar multiple transducer array (MTA). The MTA had 32 sensors in the alongshore direction and moved in the cross-shore direction to scan. We then synchronized the TLS and MTA scans to obtain the entire nearshore-beach-dune profile and calculated the average root mean square (RMS) co-registration error (BD = ±0.0128 m, VD = ±0.0192 m). For VD only, an additional scan was conducted at 0.005 m to map the plant architecture at the beginning of the experiment.

For calculating dune profile changes, we quantified the elevation difference between a “pre” and “post” scan, and clipped the datasets to only those portions that were above the standing water level (to exclude the flux into and out of the subaqueous nearshore, as it would otherwise render accretion/erosion metrics meaningless in the closed system of the LWF); this meant that a “post” scan provided the lower and more seaward limit for each calculation. We also clipped the datasets to a central 1.75-m section of the flume in the alongshore direction to eliminate errors due to wave reflection and scouring around wall-mounted sensors. We observed no consistent evidence of such behavior, but chose to be conservative, nonetheless.

Before the beginning of the experiment at 0 hours, the maximum average vertical difference at a common location on the experimental dune portion of the profile was 0.031 m (fig. S7). By 2 hours into the storm conditions, the berm on the backbeach had eroded for both VD and BD, and by 3.2 hours, their foreshore slope in front of the dunes was nearly identical, at ~9% grade. To ensure this, we selected TLS points at the base of the beach area (approximately 65.2 m in the cross-shore direction) and at the dune toe (approximately 69.7 m in the cross-shore direction), and calculated the beach slope using the equation

$$\mathrm{slope}=\frac{z_{2}-z_{1}}{x_{2}-x_{1}}$$ (1)

such that *x*_1_ and *z*_1_ were the cross-shore location and height recorded at the base of the subaerial beach, and *x*_2_ and *z*_2_ were those recorded at the subaerial dune toe. Among several thousand combinations of points, the average calculated slopes were 8.98% and 9.22% for BD and VD, respectively.

Immediately before interception at the foot of the dune by waves at 4.0 hours, we also calculated the slopes in the area where the vegetation began (at 72.5 m). We measured this slope over a 1-m distance, centered on a plant location, between 72 and 73 m and found grades of ~5 to 6% for both VD and BD. Later on at 11.3 hours, yet further up on the slope where the initial scarps later formed at 76.5 to 77.5 m, we found grades of ~14 to 15% (just before wave run-up began to hit these locations). Thousands of TLS points were selected from within 0.5 m upslope and downslope of a central location, and then the averaged slope was calculated using the equation above. Slopes within ±2% of these values were found if the cross-shore dimension was expanded to up to 3 m in length.

### Wave run-up and dune micro-slopes

We tracked run-up and swash events, individual bores, and short-time scale accretion/erosion continuously, using a Riegl Z390i line-scan TLS (Riegl Laser Measurement Systems GmbH). The scanner recorded the bare and inundated profile heights at a 2-Hz sampling rate, and 0.006 m accuracy, along a single transect at the center of the flume. We co-registered the data using a plane rectification method (*35*). We then determined the elevation of the sand in the swash zone by using a 25-s moving minimum filter and subtracted its height from the original data to calculate the inundation depth. This procedure also automatically removed the vegetation from the scene below *R*_max_, which we cross-checked against the manually cleaned Leica TLS data, finding similar results. Finally, we defined the maximum run-up distance (*R*_max_) as the farthest point landward where the inundated depth was <0.02 m (we could not reliably detect run-up below this height). The *R*_2%_ distance was calculated as the cross-shore location of the elevation that was exceeded by only the largest 2% of run-up bores.

Dune micro-slopes were identified by calculating the average slope in the cross-shore direction within 0.2-m bins at 2-Hz sampling rate and 0.006-m accuracy, as mentioned above, using the Riegl line-scan data.

### Dune volumetric water content

The volumetric water content of the dune sands (figs. S12 and S13) was recorded at a wide variety of depths and cross-shore locations (*36*). We used an array of 24 Teros-10 sensors and calibrated them using dune sand samples at six different water contents, ranging from air-dried sand to the highest possible saturation. The absolute readings from the sensors were a function of both the void ratio of the sand (volume of empty spaces relative to volume of solids, whether filled with air or water) and the water within the voids. In the event that either varied, the measure quantified the volume of water per volume of sand. The excess volumetric water content at a cross-shore location was calculated as the difference between the absolute readings near the sand surface, at a depth of 0.15 m, and the absolute readings at lower depths inside the dune (at 0. 65 m of depth for the vertical stack at 73 m, and 0.68 m for the 75 m; Fig. 3 and fig. S12), divided by the readings near the sand surface times 100. This excess value measured the relative change in the surface compared to the depth over time. We note that by standardizing the absolute surface readings by depth readings, we factor out initial void ratio differences by cross-shore location and dune type, allowing us to focus on the changes in the excess value over time relative to their initial value.

We found that the volumetric water content at the near surface increased as run-up inundated the cross-shore position of the sensors (fig. S13). Early on, VD volumetric water content was generally lower than BD, as it had not been inundated by run-up for as much time. However, once a critical point was reached in the morphologic sequence (see main text), VD became supersaturated and the volumetric water content abruptly increased (for example, at ~12.5 hours at 73 m in fig. S13). The sensor shortly thereafter eroded from the dune (as indicated by the vertical spike and drop). Erosion at each location occurred for VD before BD.

A predisposition to instability appeared correlated with ~40 to 50% volumetric water content at the near surface (fig. S13) and in particular when the near surface reading exceeded that at depth (Fig. 3). As the sequence of events progressed further in time, and the volumetric water content exceeded ~50% at the near surface (fig. S13), the excess volumetric water content became even more strongly positive (Fig. 3) and excess pore pressure gradients between the near-surface and lower depths also rapidly increased [and as measured using 15 Druck CDR 1830 pressure sensors, colocated with the volumetric water content sensors at most locations in fig. S12; see (*36*)]. For a more detailed description of the sequence of events in terms of inundation by run-up bores, increases in volumetric water content, increases in pore pressure, the transmission of pressure waves through the sand medium, partial momentary liquefaction, and dune instability, we refer the reader to (*36*).

### Wave reflection

Wave reflection was assessed using the free-water surface elevations from four wave gauges at cross-shore locations 28.737, 29.659, 31.201, and 32.421 m, and then the reflection coefficient, Kr, was calculated as

$$\mathrm{Kr}=H_{r}/H_{i}$$ (2)

where the incident significant wave height *H*_i_ and the reflected significant wave height *H*_r_ waves were estimated using the algorithm for nonlinear waves within the WaveLab program (*37*–*38*) and then averaged for each trial.

We calculated the Iribarren number, ξ, following the standard formula

$$\xi =\frac{\tan\;\alpha }{\sqrt{H_{i}/L_{0}}}$$ (3)

where tan α is the rise divided by the run, or the slope as given by the equation in the above section on the profile geometry; *H*_i_ is the incident significant wave height and *L*_0_ is the deep water wavelength such that $L_{0}=\frac{g}{2\pi }T_{p}^{2}$, given *g* as gravitational acceleration and *T*_p_ as the recorded peak wave period during the trial.
