# Supplementary material for: Does vegetation accelerate coastal dune erosion during extreme events?
Source: Sci Adv. 2023 Jun 14;9(24):eadg7135. doi: 10.1126/sciadv.adg7135 (PMC10266738; doi:10.1126/sciadv.adg7135)
Supplement: Supplementary file 1 — Figs. S1 to S13 Table S1 Legends for movies S1 and S2 [file sciadv.adg7135_sm.pdf]

Science Advances
AAAS

Supplementary Materials for

Does vegetation accelerate coastal dune erosion during extreme events?

Rusty A. Feagin *et al.*

Corresponding author: Rusty A. Feagin, feaginr@tamu.edu

Sci. Adv. **9**, eadg7135 (2023)
DOI: 10.1126/sciadv.adg7135

The PDF file includes:

Figs. S1 to S13
Table S1
Legends for movies S1 and S2

Other Supplementary Material for this manuscript includes the following:

Movies S1 and S2

Fig. S1. The hydrodynamic conditions during Hurricane Sandy, offshore of Mantoloking, New Jersey.

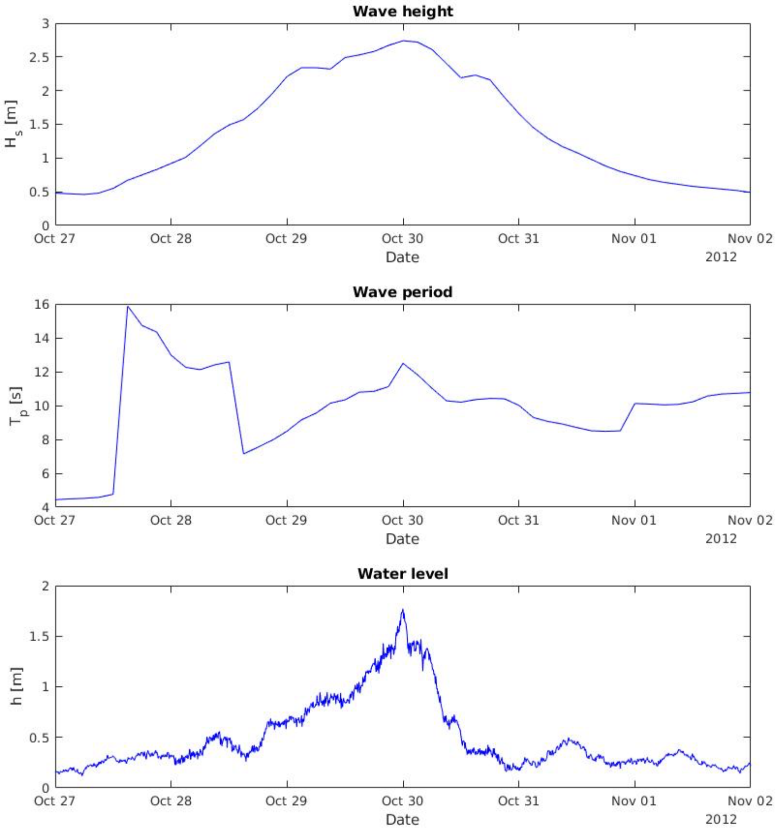

Fig. S2. The designed hydrodynamic conditions for the Long Wave Flume (LWF) experiments in Corvallis, Oregon, and those during Hurricane Sandy, offshore of Mantoloking, New Jersey (re-scaled at 2:25 to 1). The experiment stopped at the peak of the storm conditions.

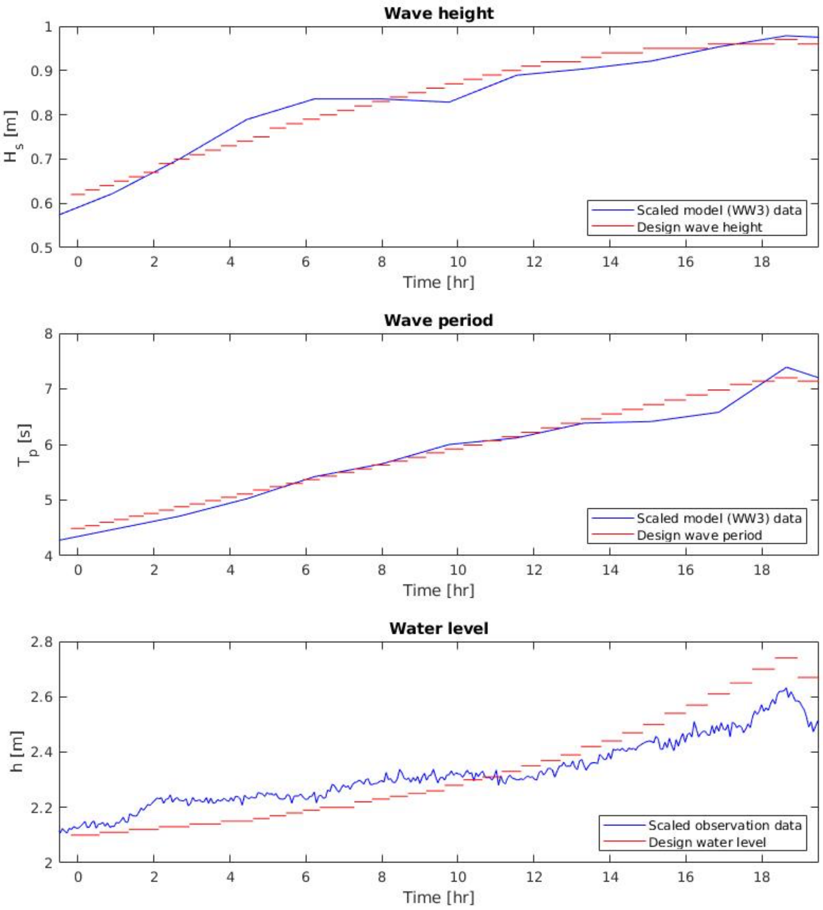

Fig. S3. **Dune profile and instrumentation plan.** Axes not to scale. Both the vegetated (VD) and bare (BD) dunes followed this set up.

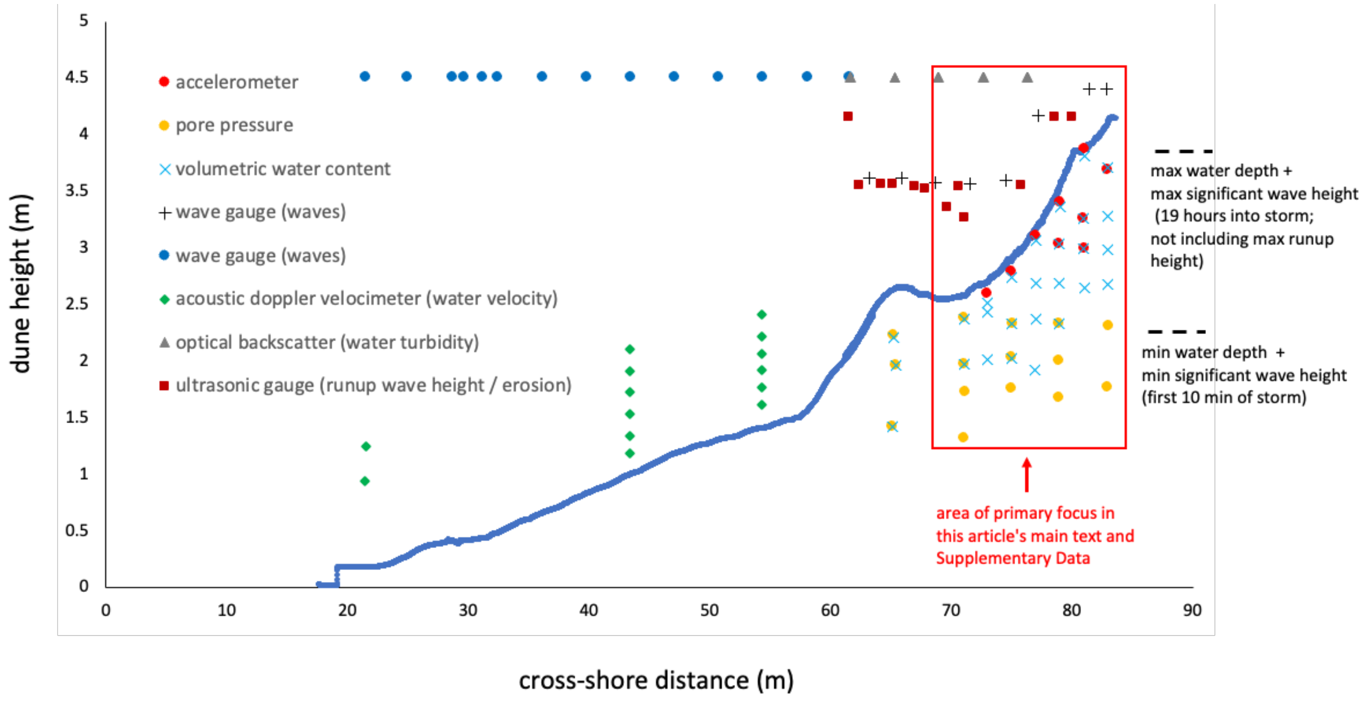

Fig. S4. Terrestrial Laser Scanning (TLS) data of the VD dune profile, inside the wave flume. Horizontal and vertical are to proper scale relative to one another. Vertical lines on the left of image are the flume walls. The profile can be seen from bottom left corner of the image and extending to the middle portion on the right of image (vegetation is barely visible on top of profile on right).

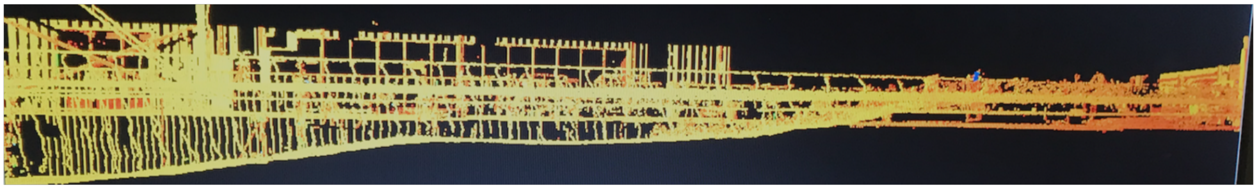

Fig. S5. Dune sand was obtained from South Beach, Newport, Oregon.

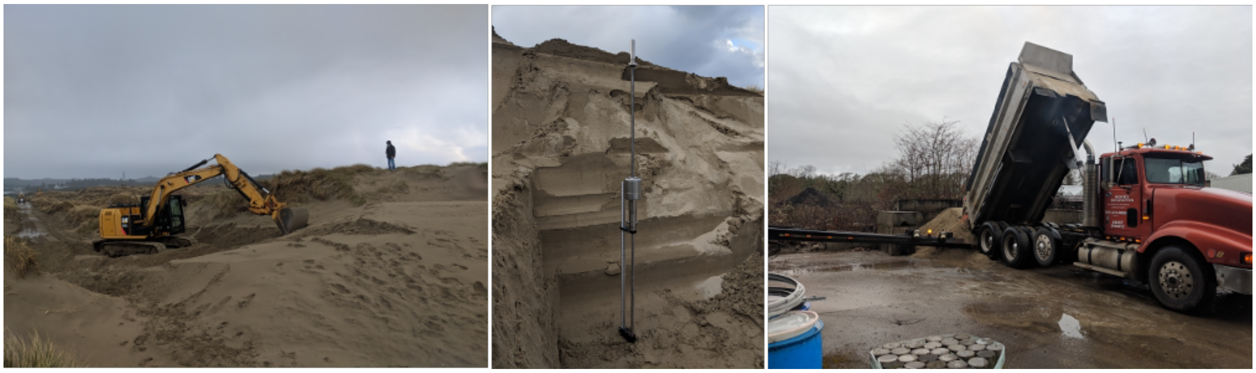

Fig. S6. The dune sand was placed in the LWF in Corvallis, Oregon.

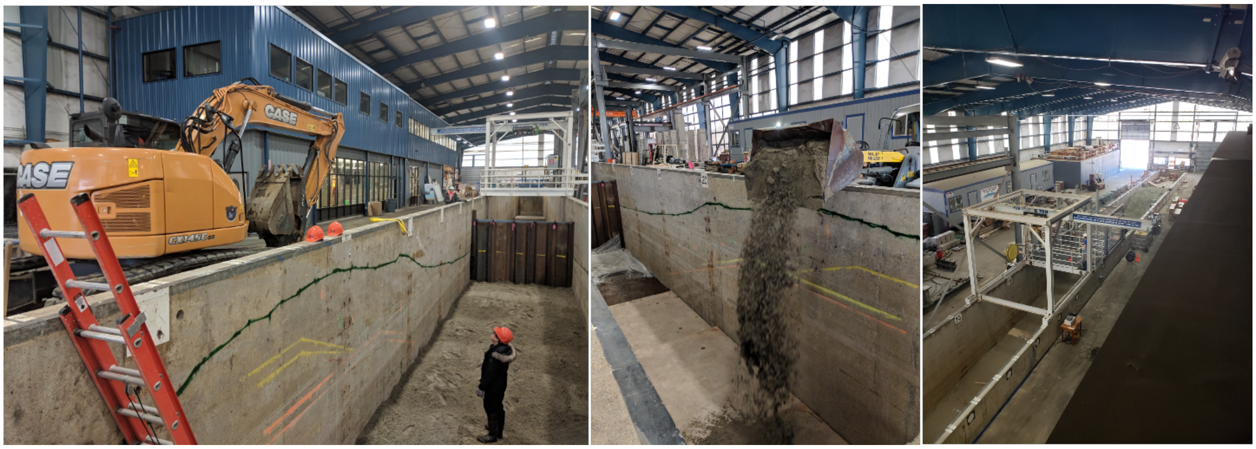

Fig. S7. At the beginning of the experiment, the dune profile and sediment compaction were similar between the VD and BD dunes. Part (a) shows the entire profile, part (b) shows the vegetated portion of the cross-shore profile from 72 to 84 m. Part (b) also shows compaction data as collected by dynamic cone penetrometer.

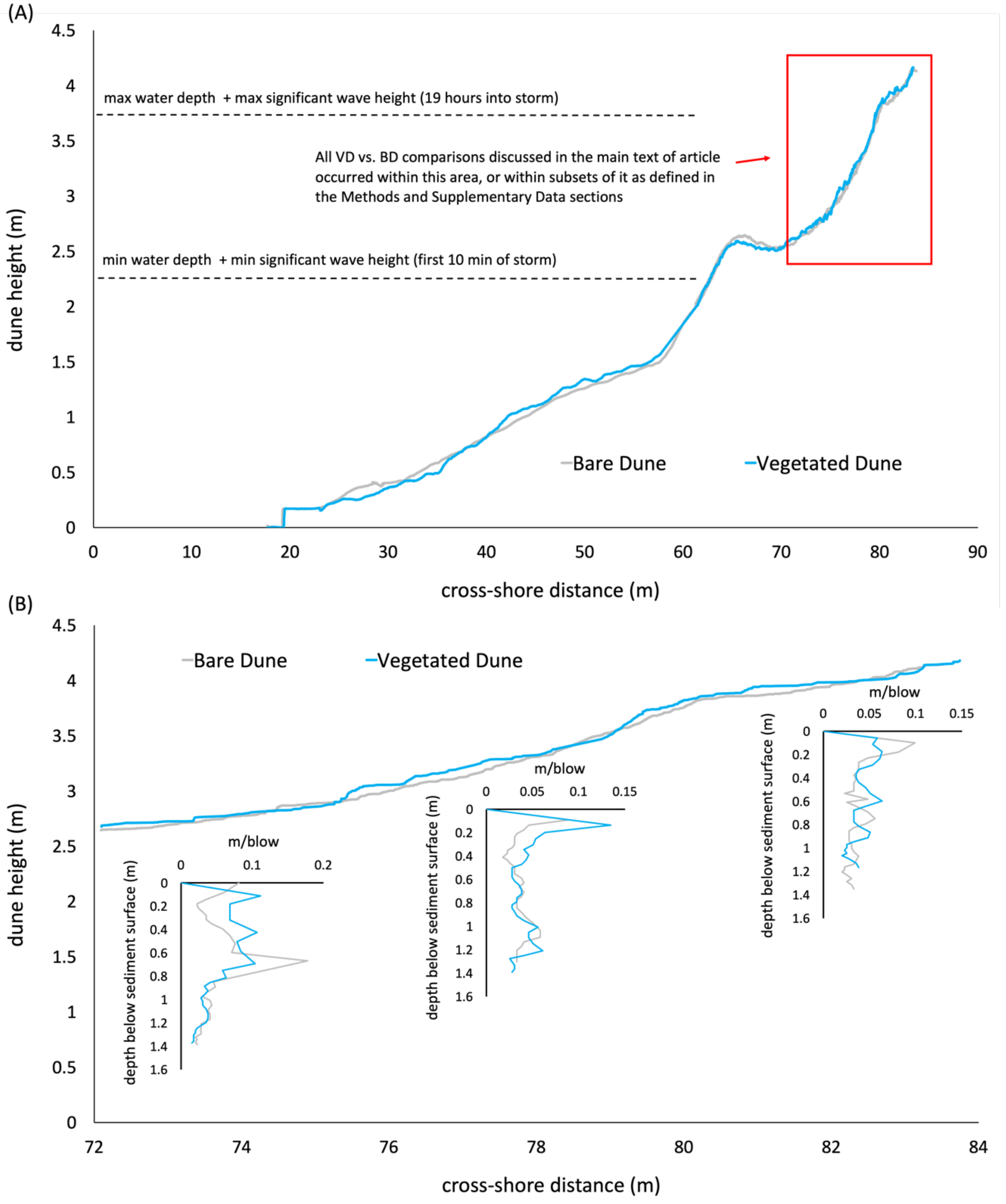

Fig. S8. The greenhouse set-up in the LWF facility, placed over one end of the 104 m long flume.

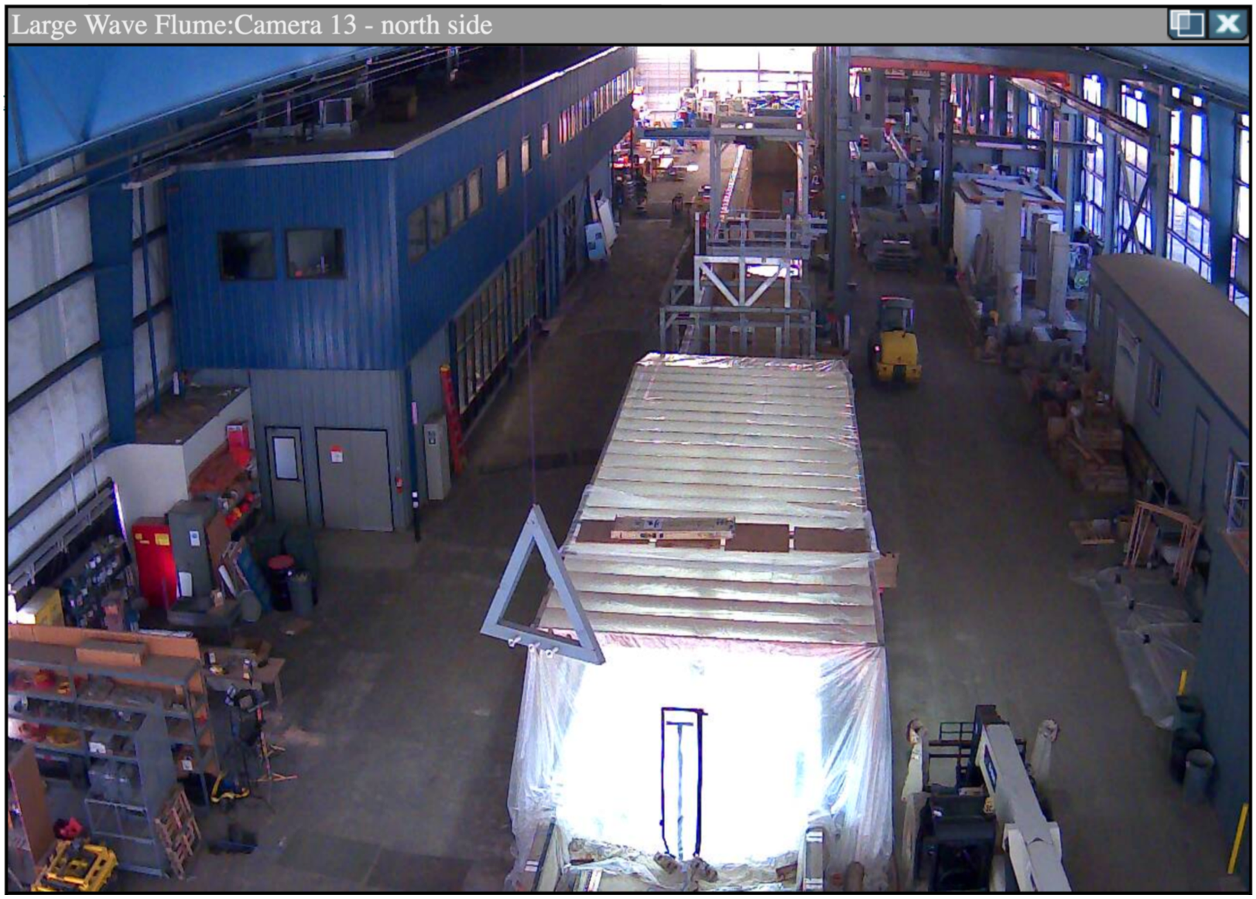

Fig. S9. Inside the greenhouse after ~2 months while the plants were growing (top), and after 6 months after greenhouse removal (bottom).

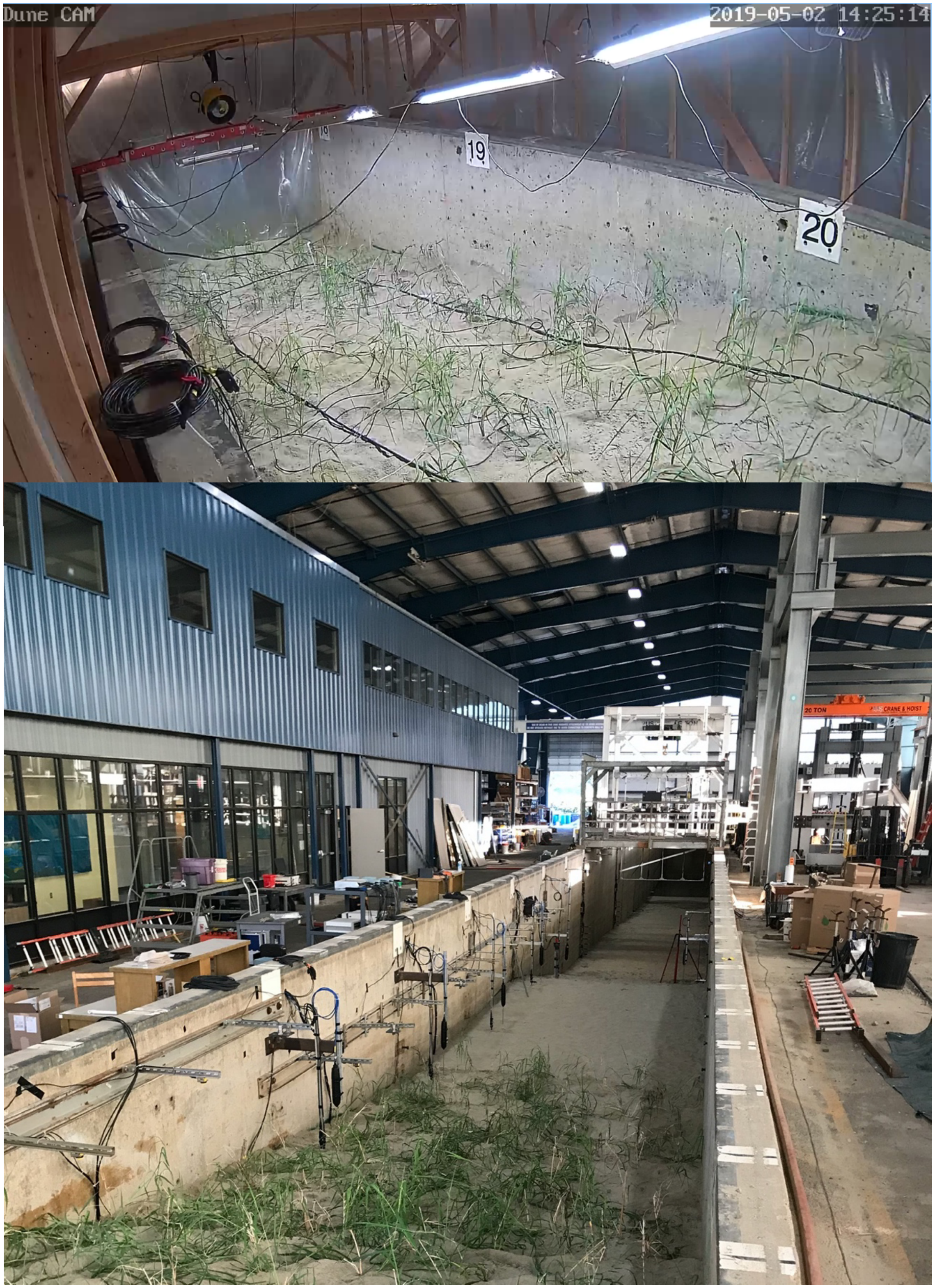

Fig. S10. ***Panicum amarum*** **plants in VD during wave interception of the scarp, around 18.4 hours into the experiment.**

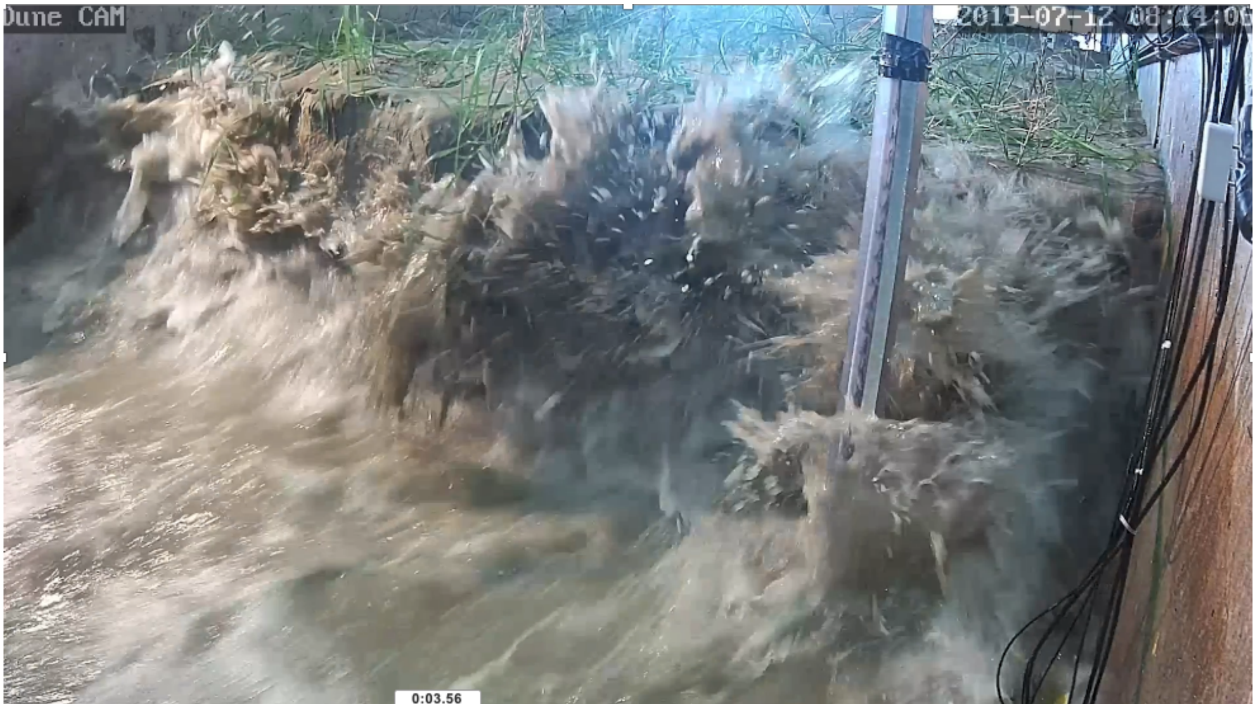

Fig. S11. Example of TLS scan, viewed from side. Red points are from the 0.005 m scan taken before the experiment. The other color points represent dune profile elevations after the vegetation points were extracted from the dataset, from times between 10.3 and 12.3 hours into the storm.

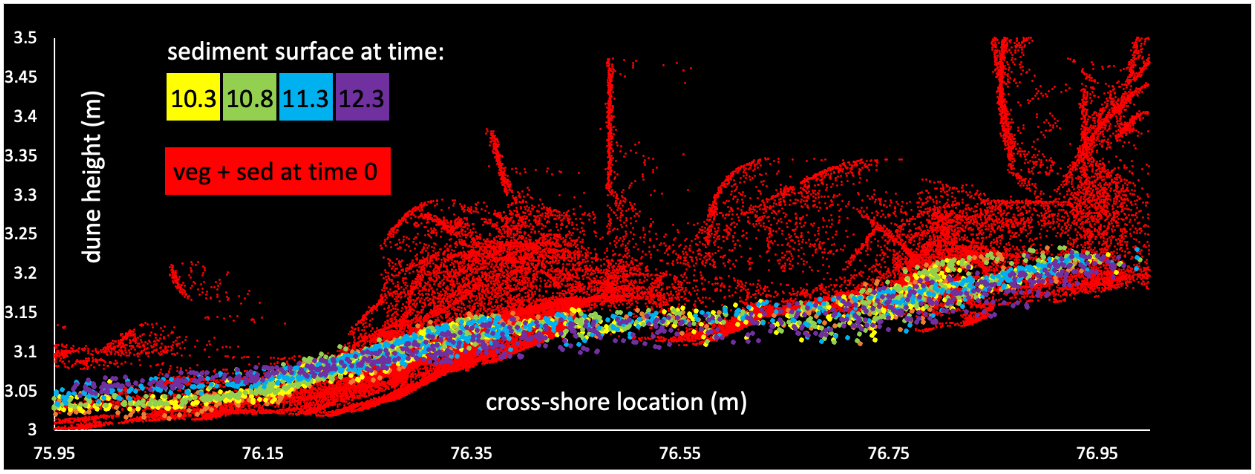

Fig. S12. Locations of the volumetric water content sensors in the vegetated dune VD. Sensors used for calculating the excess volumetric water content presented in Fig. 3 of the main text included #8 and #6 (73 m cross-shore), and #11 and #9 (75 m cross-shore). Sensors depicted in Fig. S13 included #8 (73 m), #11 (75 m), and #14 (77 m).

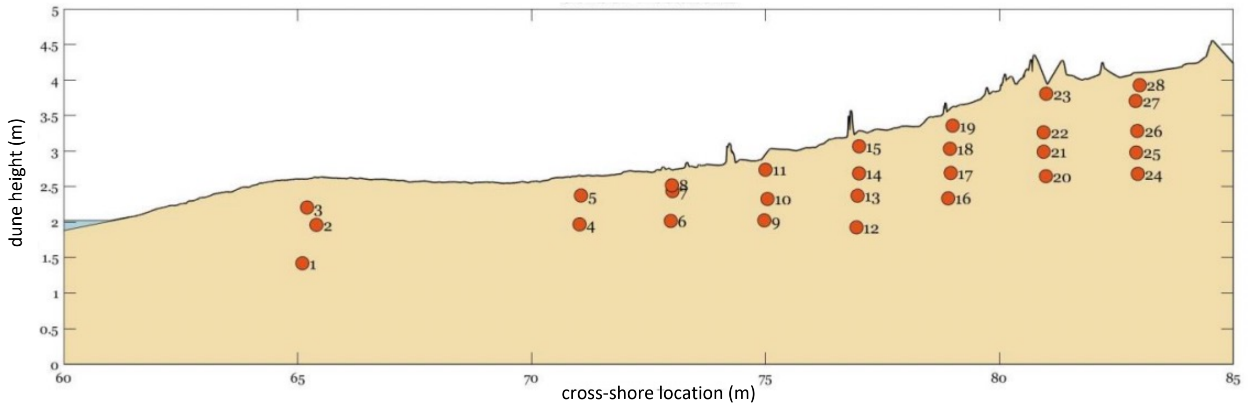

Fig. S13. The volumetric water content of VD versus BD over time, at 73, 75, and 77 m in the cross-shore direction. All sensors shown here were located 0.15 below the immediate dune surface (dune height locations are at 2.515, 2.735, and 3.065 m, respectively).

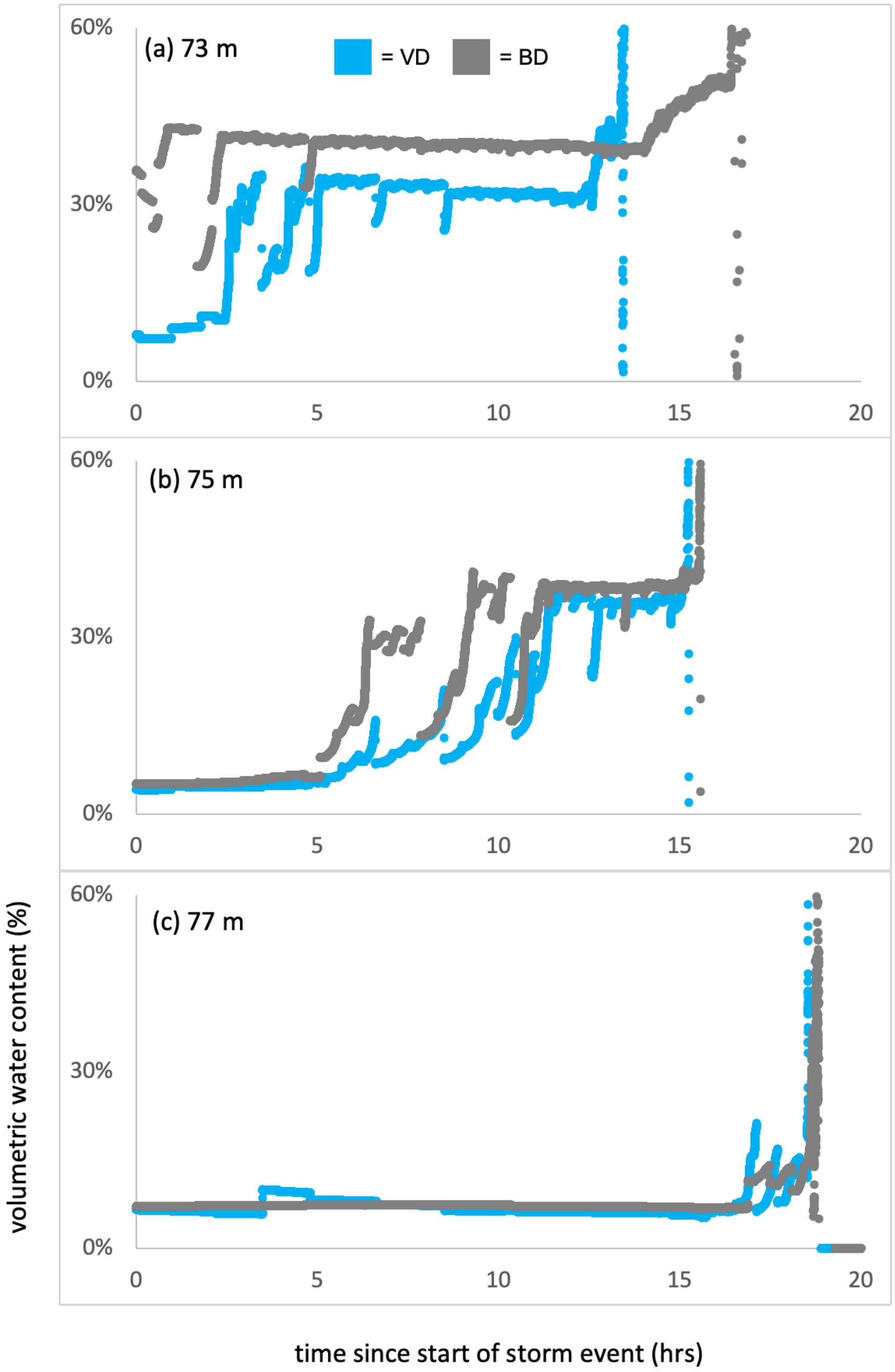

Table S1. Dimensionless parameters for scaling processes of the dune experiment.

	Parameter	Ratio: field/lab
**Profile Geometric Scaling**	h, L	$\frac{h_{field}}{h_{lab}} = \frac{L_{field}}{L_{lab}} = 1$
**Reynolds #**	$Re = \frac{Uh}{\upsilon}$	$\frac{Re_{field}}{Re_{lab}} = 1.4$
**Shields #**	$\theta = \frac{u_*^2}{(s-1)gd_{50}}$	$\frac{\theta_{field}}{\theta_{lab}} = 2.25$
**Froude #**	$Fr = \frac{L}{T\sqrt{gh}}$	$\frac{Fr_{field}}{Fr_{lab}} = 1$
**Vegetation Drag Coefficient**	$C'_D = C_D\left(\frac{\ell p}{\Gamma h}\right)$	$\frac{C'_{D,field}}{C'_{D,lab}} = 1$

Movie S1. Overview of the vegetated dune (VD) prior to the experiment, in the LWF flume in Corvallis, Oregon. (See online at *Science Advances*).

Movie S2. Example of 0.005 m resolution Terrestrial Laser Scanning (TLS) imagery of plant structure on the vegetated dune (VD) prior to the experiment. (See online at *Science Advances*).
